# A Review on the Design of Hydrogels With Different Stiffness and Their Effects on Tissue Repair

**DOI:** 10.3389/fbioe.2022.817391

**Published:** 2022-01-25

**Authors:** Tianyi Luo, Bowen Tan, Lengjing Zhu, Yating Wang, Jinfeng Liao

**Affiliations:** ^1^ State Key Laboratory of Oral Diseases, National Clinical Research Centre for Oral Diseases, West China Hospital of Stomatology, Sichuan University, Chengdu, China; ^2^ West China School of Pharmacy, Sichuan University, Chengdu, China

**Keywords:** hydrogel, stiffness, tissue repair, stem cell, macrophage, inflammatory response

## Abstract

Tissue repair after trauma and infection has always been a difficult problem in regenerative medicine. Hydrogels have become one of the most important scaffolds for tissue engineering due to their biocompatibility, biodegradability and water solubility. Especially, the stiffness of hydrogels is a key factor, which influence the morphology of mesenchymal stem cells (MSCs) and their differentiation. The researches on this point are meaningful to the field of tissue engineering. Herein, this review focus on the design of hydrogels with different stiffness and their effects on the behavior of MSCs. In addition, the effect of hydrogel stiffness on the phenotype of macrophages is introduced, and then the relationship between the phenotype changes of macrophages on inflammatory response and tissue repair is discussed. Finally, the future application of hydrogels with a certain stiffness in regenerative medicine and tissue engineering has been prospected.

## Introduction

Trauma, infection, tumor, and other diseases can cause tissue damage and have an adverse impact on physiological function (Wang et al., 2018; Esser-von Bieren, 2019). Tissue repair mainly includes bone/cartilage tissue defect repair and ordinary wound healing. The repair process involves the recruitment of related cells, protein secretion, and cytokine expression (Fu et al., 2019; Ghilardi et al., 2020). Traditional treatment methods have the disadvantages of causing immune rejection or lack of implant donors (Li et al., 2018; Yushan et al., 2020). Some treatment methods may also adversely affect the patient’s body and mind (Yushan et al., 2020). Therefore, it is necessary to take appropriate measures for tissue repair to reduce the damage to the body.

Recently, the emerging interdisciplinary-tissue engineering integrating materials science and cell biology has made it hopeful to solve the problems of tissue repair. The three elements of tissue engineering include scaffold materials, cytokines, and stem cells. As one of the scaffold materials, hydrogel plays an important role in tissue engineering because of its high water content, hydrophilicity, simulation of extracellular matrix environment (Abdollahiyan et al., 2020a; Liao et al., 2021a; Liao et al., 2021b; Tan et al., 2021a), good biocompatibility, and the ability to control drug release (Gao et al., 2016; Huang et al., 2017; Tan et al., 2021b). It is a hydrophilic three-dimensional (3D) polymer network formed by physical or chemical crosslinking. And it have been increasingly used for tissue repair recently, such as wound healing, bone tissue repair, etc. Some silk-elastic protein hydrogels were used for wound healing because they can change from the solution state to the gel state at physiological temperature and maintain the moisture in the wound (Wen et al., 2020). In addition, some self-healing hydrogels have antibacterial and anti-infection properties. and they were used for wound repair by carrying related drugs (Zhao et al., 2017; Shen et al., 2021). At the same time, the method of adapting hydrogel to the bone tissue environment by changing its stiffness has also been increasingly applied in bone tissue engineering (Dubey et al., 2020; Ji et al., 2020). Recent studies have found that the growth, reproduction, and differentiation of cells can be manipulated by changing the physical properties of hydrogels, thereby affecting the repair process of bone tissue (such as the stiffness, mechanical strength, surface charge, and other physical factors of extracellular matrix) (Trappmann et al., 2012; Mills et al., 2020; Mohammed et al., 2021). Most studies obtained hydrogels with different stiffness by changing the external stimuli or the composition of hydrogels. These hydrogels with stiffness changes have also been widely used in the study of cell behavior changes. For example, increased hydrogel stiffness can induce human mesenchymal stem cells to differentiate into cartilage, making it a potential candidate for cartilage regeneration medicine (Wang et al., 2020a; Mohammed et al., 2021). In addition, hydrogels can also be used for tissue repair by affecting redox reactions. According to relevant experimental reports, polyacrylamide (PAAm) hydrogel with kPa stiffness can make MSCs express more reactive oxygen species (ROS) than traditional fibronectin (FN) coating hydrogel with GPa stiffness (Yang et al., 2016). The increased expression of ROS can increase the content of MSCs related protein secretome and regulate the related physiological processes in the process of tissue repair. In addition, some hydrogel-based composite platforms can also change the phenotype of macrophages by increasing the hydrogel stiffness, transforming them from anti-inflammatory M2 type to pro-inflammatory M1 type, and promoting tissue repair by regulating related inflammatory reactions and immune processes (Meli et al., 2020; Yuan et al., 2021). In general, it can affect the reproductive differentiation or phenotype of cells by changing the related biophysical properties of hydrogels and affecting the expression of related cytokines or protein secretion groups.

Stem cells are a kind of cells with a strong ability of division and differentiation and long-term self-renewal. Under certain conditions, it can differentiate into chondrocytes, osteoblasts, fibroblasts, bone cells, and other cells that play an important role in hard/soft tissue repair and enhance the proliferation ability of related cells (Chan et al., 2015; Paschos et al., 2015; Walmsley et al., 2016). According to their differentiation ability, they can be divided into totipotent stem cells, pluripotent stem cells, multipotent stem cells, oligopotent stem cells and unipotent stem cells (Zakrzewski et al., 2019). The role of MSCs in the body is not versatile, so it does not belong to the category of stem cells. Related studies have shown that MSCs originate from peripheral cells and are a kind of stromal cells differentiated *in vitro* (Caplan, 2017). For MSCs, it can be induced to differentiate into related components in the bone marrow microenvironment such as bone marrow and fat, and are often used as seed cells for tissue repair in ideal cases (Bartholomew et al., 2002). In the early stage of bone defect repair, inflammatory reactions often occur in the defect site and release related inflammatory factors (Bahney et al., 2019) while MSCs play a significant role in the process of bone repair due to their characteristics of immune regulation. When inflammation occurs, immune cells produce related cytokines, which attract bone marrow mesenchymal stem cells (BMSCs) to the defect area, and then MSCs differentiate into osteoblasts (Simon et al., 2002; Spaeth et al., 2008; Bahney et al., 2019) to promote bone defect repair.

Both stem cells and MSCs are affected by microenvironment, the clues in the microenvironment also play a very important role in the life activities of cells. The factors like microgravity (Chen et al., 2016; Chen et al., 2017), cell-cell contact (Sun et al., 2012), matrix stiffness affect the growth, reproduction, and differentiation of cells. Among them, matrix stiffness can affect cell differentiation and phenotype by affecting the expression of cytokines, ROS expression, the secretion of protein components, thus stiffness play a crucial role in tissue defect repair. Recently, adjusting the mechanical properties of hydrogels on cell differentiation lineage has become a research hotspot (Ma et al., 2017; Lee et al., 2018a; Baruffaldi et al., 2021). Specifically, the increase of hydrogel matrix stiffness MSCs cells differentiate into osteoblasts and the cell colonies on the matrix surface are more dispersed. On hydrogels with low matrix stiffness, stem cells tended to differentiate into cartilage or fat, and cell colonies showed a more rounded shape. Among them, the change of hydrogel stiffness may affect the phenotype of stem cells through the expression of related genes and proteins (Wen et al., 2019; Phuagkhaopong et al., 2021). On the surface of the hydrogel with higher stiffness, the nuclear-cytoplasmic ratio of Yes-associated-protein (YAP)/transcriptional coactivator with PDZ-binding motif (TAZ) is higher, resulting in osteogenic differentiation. On the surface of the scaffold with lower stiffness, the nuclear-cytoplasmic ratio of YAP/TAZ is lower, which makes the cells differentiate into adipocytes (Stanton et al., 2019a; Cao et al., 2019).

The immune system is an important part of the repair process of tissue injury. After tissue injury, it lead to the proliferation and differentiation of some immune cells and release related cytokines to the injury site. Macrophages are one of these cells. Macrophages have two different phenotypes, M1 (pro-inflammatory) and M2 (pro-healing). M1 releases pro-inflammatory cytokines, while M2 can promote tissue repair and down-regulate inflammatory response (Chung et al., 2017). In the inflammatory response after bone repair immune response, natural killer (NK) cells and macrophages can enhance MSC migration by secreting Chemokine (C-X-C motif) ligand 7 (CXCL7) or chemokines, respectively (Liu et al., 2018). Relevant studies have also shown that macrophages can promote bone differentiation of MSC through interleukin (IL)-23 cytokines secreted by macrophages in the inflammatory environment (Tu et al., 2015). The change of macrophage phenotype is related to matrix stiffness. On the surface of the high stiffness matrix, macrophages are pro-inflammatory phenotype, while on the surface of low stiffness matrix, macrophages are anti-inflammatory phenotype (Trel’ová et al., 2019; Taskin et al., 2021). Changes in macrophage phenotypes, in turn, alter the expression of related cytokines, which can affect the bone microenvironment (YlÖstalo et al., 2012; Liu et al., 2018; Esser-von Bieren, 2019). Second, different macrophage phenotypes affect BSCs behavior, thereby affecting endothelial cell angiogenesis and promoting tissue repair (Liao et al., 2019).

In this review, we introduced the fabrication of hydrogels with different stiffness and the effects of related biophysical cues on cell differentiation and proliferation. At the same time, we described the research of different hydrogels in tissue repair and osteogenic differentiation. We hope to provide new research ideas for regenerative medicine and tissue engineering by analyzing the mechanism of action of different stiffness hydrogels on cells.

## The Fabrication Methods of Hydrogels With Different Stiffness

Biomaterials with different matrix stiffness have been widely used to study cell differentiation and phenotype (Cai et al., 2020; Mohammed et al., 2021). At the same time, the related research of hydrogels with different stiffness in tissue repair has become a research hotspot. Stiffness-tunable hydrogels can be obtained by changing the crosslinking degree of gels, stimulating external conditions, changing the molecular weight of materials, and adding nanomaterials. Based on different experimental designs, hydrogels with different characteristics and different stiffness can be obtained. Table 1 summarizes various hydrogel design methods and corresponding stiffness. The following sections will introduce the design methods of hydrogels with different stiffness.

**TABLE 1:** Summary of the hydrogels with different stiffness by different design methods.

Methods	Hydrogels	Parameter	Strength	Ref
Changing the crosslinking of hydrogel	Methacrylated gelatin (GelMA)/methacrylated chitosan (CSMA)/polyhedral oligomeric silsesquioxane (POSS)	365 nm light	108 kPa	Zhang et al. (2019)
	Curdlan/HPAAm (H stands for the hydrophobic association)	1)Chemically crosslinked PAAm	1)12 kPa	Ye et al. (2020)
		2)Physically crosslinked HPAAm	2)49 kPa	
		3)Physically–chemically crosslinked curdlan/PAAm double network (DN) gel	3)103 kPa	
	Alginate	The volume of crosslinking agent (CaCl_2_)	80.25 kPa (1 ml)	Naghieh et al. (2018)
			99.3 kPa (3 ml)	
	1 Polyvinyl alcohol (PVA) single network (SN)	Single/double network crosslinking	1,124 kPa	Sabzi et al. (2017)
	2 Agar/PVA DN		2 221 kPa	
	Polyacrylamide	The concentration of crosslinking agent diacrylamide	719 Pa (0.5 μL)	Abdallah et al. (2019)
			5.9 kPa (30 μL)	
Changing external stimuli)	Azobenzene (crosslinker)/PAAm	Ultraviolet rays (UV) light	Before UV light: 8.3 ± 2.0 kPa	Lee et al. (2018b)
			After UV light: 2.0 ± 0.6 kPa	
	Polyisocyanopeptide (PIC)-DNA	pH	35 Pa (pH = 7.4)	Deshpande et al. (2017)
			100 Pa (pH = 5.2)	
	AuNRs (crosslinker)/Poly (1-vinylimidazole-co-methacrylicacid)	Near infrared (NIR) light	Before NIR light: 13 kPa	Dai et al. (2020)
		UV light	After UV light:57 kPa	
	Poly (2-(diisopropylamino)ethyl methacrylate) (PDPA) 50 - poly (2-(methacryloyloxy)ethyl phosphorylcholine) (PMPC) 250 -PDPA 50	pH	1.4 kPa (pH = 7)	Yoshikawa et al. (2011)
			40 kPa (pH = 8)	
	Alginate-F127 (0.5A–30F)	Temperature	2,900 Pa (15°C)	Quah et al. (2020)
			40,000–50,000 Pa (25 and 40°C)	
	PAAm/carbonyl iron	magnetic field:0Tor0.75T	0.1–0.14 kPa (0T)	Abdeen et al. (2016)
			60–90 kPa (0.75T)	
Changing molecular weight of materials	N-(2-aminoethyl)maleimide/hyaluronic acid (HA)	Molecular weight of HA	HA-4 kDa ˂0.2 kPa	Ren et al. (2021)
			HA-90 kDa˃ 1 kPa	
	chondroitin sulfate (CS)/HA	Molecular weight of HA	G′ (10 kD)<G′ (60kD)	Nguyen et al. (2021)
	Vitamin E (VitE) 1.18 -PEG20K-VitE 1.18	Molecular weight (PEG)	680 Pa (10,000 Da)	Yang et al. (2019a)
			2,611 Pa (20,000 Da)	
Changing the proportions of components	FN/PAAm	Acrylamide/bis-acrylamide = 4: 0.15 or 10:0.1	1.5 kPa (4:0.15)	Yang et al. (2016)
			17.7 kPa (10:0.1)	
	PAAm	Acrylamide/bis-acrylamide = 15%: 1% or 8%:0.48%	68 kPa/mm (15%: 1%)	Sunyer et al. (2012)
			7.5 kPa/mm (8%: 0.48%)	
	Graphene oxide (GO)/PAAm	Acrylamide/bis-acrylamide = 3: 0.1 or 10:0.3	2 kPa (3:0.1)	Sun et al. (2018)
			32 kPa (10:0.3)	
Adding nanomaterials	Nanosilicate/collagen	Nanosilicate: 0%, 0.5%, 1%, 2%	3.3 ± 0.4 kPa (0%)	Xavier et al. (2015)
			4.7 ± 0.9 kPa (0.5%)	
			8.9 ± 2.1 kPa (1%)	
			12.9 ± 1.3 kPa (2%)	
	Whitlockite (WH)/hydroxyapatite (HAP)/GelMA	HAP: 1 μg/ml-1000 μg/ml	GelMA 23 kPa	Cheng et al. (2018)
		WH: 1 μg/ml-1000 μg/ml	HAP/GelMA 23–29 kPa	
			WH/GelMA 18–24 kPa	
	GO/PAAm	GO	PAAm: 18 ± 1 kPa	Jo et al. (2017)
			GO/PAAm: 54 ± 10 kPa	
	Nanosilicates/gelatin methacryloyl (GelMA)/methacrylated kappa carrageenan (MkCA)	Nanosilicates: 0 %wt/v or 0.5% wt/v	3.5 ± 0.6 kPa (0 % wt/v)	Cross et al. (2018)
			5.9 ± 1.8 kPa (0.5% wt/v)	
	(Carboxymethyl chitosan)CMC/GO-CMC	GO	4.99 ± 0.02 kPa (CMC)	Zou et al. (2021)
			5.62 ± 0.03 kPa (GO-CMC)	

### Regulating the Stiffness of Hydrogels by Changing the Crosslinking Way of Hydrogel

Biomaterials or synthetic materials can form hydrogels by different crosslinking methods (Preethi Soundarya et al., 2018). Crosslinking methods, the amount of crosslinking agent, and different crosslinking conditions affect the physical properties of the hydrogel, especially its stiffness. In Di Giuseppe’s experiment, they prepared a series of gradient concentrations of alginate and gelatin and synthesized hydrogels in the form of ionic crosslinking by changing the crosslinking time. We know that the conventional preparation method of alginate hydrogel is through Ca^2+^ ion crosslinking, and hydrogels with different crosslinking degrees can be obtained by changing the crosslinking time and temperature. In this study, the results showed that the stiffness of the hydrogel was significantly improved at high concentrations and long crosslinking time (Giuseppe et al., 2018). In previously reported DN hydrogels (Haraguchi and Takehisa, 2002; Jian, 2010; Gu et al., 2018; Zhao et al., 2019), due to the formation of double network structure, the second layer material played a role of scaffold to maintain the configuration of DN, thereby greatly improving mechanical properties of hydrogels. The first network of the DN hydrogel is composed of hard and brittle polyelectrolytes, which is a tightly connected structure; the second network is composed of a soft and charge-free polymer, which is a loose structure (Gu et al., 2018; Zhao et al., 2019). Thus the stiffness of hydrogels can be changed by the composition or crosslinking mode of these two networks. Li et al. (2020) prepared gellan gum (GG)/polyethylene glycol diacrylate (PEGDA) DN hydrogels by physical crosslinking and chemical crosslinking of GG and PEGDA, respectively. They tested their mechanical properties and their effects on BMSCs activity. The experimental results showed that the GG/PEGDA DN hydrogel has good ductility compared with the single uncrosslinked hydrogel (Li et al., 2020). Sabzia et al. (2017) recently designed a double physical crosslinking Agar/polyvinyl alcohol (Agar/PVA) DN hydrogel. Compared with mixed crosslinking or pure chemical crosslinking, Agar/PVA DN hydrogel demonstrated enhanced mechanical properties (Sabzi et al., 2017). At the same time, double physical crosslinking hydrogel also showed good self-healing properties (Sabzi et al., 2017; Ye et al., 2020). Some studies have reported that degradable DN hydrogels are used to induce cartilage differentiation. Zhang et al. (2019) prepared a mixed DN hydrogel by two-step photo-crosslinking. Firstly, different concentrations of Octamethacrylated polyhedral oligomeric silsesquioxane (OMAPOSS) and fixed concentrations of methacrylated chitosan (CSMA)-sodium dodecyl sulfate (SDS) and lithium phenyl-2,4,6-trimethyl-benzoyl phosphinate (LAP) were dissolved in dimethyl sulfoxide (DMSO) to obtain nano-hybrid hydrogels with different concentrations. Then, the hydrogels were irradiated with light at 365 nm light for 10 min to complete the first step of crosslinking. The second step is to dissolve the pre-prepared chitosan (CS)-POSS hydrogel in GelMA and LAP solution and irradiate 1 min with 365 nm light. It is because of the formation of the interconnection network that the structure of the composite hydrogel is more compact. The stiffness was improved, which also enhances the osteogenic differentiation ability of MSCs (Zhang et al., 2019). In addition, the stiffness of hydrogels can also be changed by changing the formation of the crosslinking network of hydrogels. Schweller and West (Schweller and West, 2015) designed a peptide sequence modified by Lys [allyloxycarbonyl (alloc)] amino acid for the construction of hydrogels. It was found that compared with PEG (Poly (ethylene glycol))-PQ (GGGGGPQGIWGQGGGGK peptide sequence) hydrogel, PEG-PQ (alloc) hydrogel was mainly in the form of single-molecule connection, resulting in the decrease of crosslinking degree and hydrogel stiffness. In addition to affecting the osteogenic process, the softer PEG-PQ (alloc) hydrogel had a positive effect on promoting the formation of tissue blood vessels, which is be conducive to future tissue repair treatment.

### Regulating the Stiffness of Hydrogels by Changing External Stimuli

**FIGURE 1:**
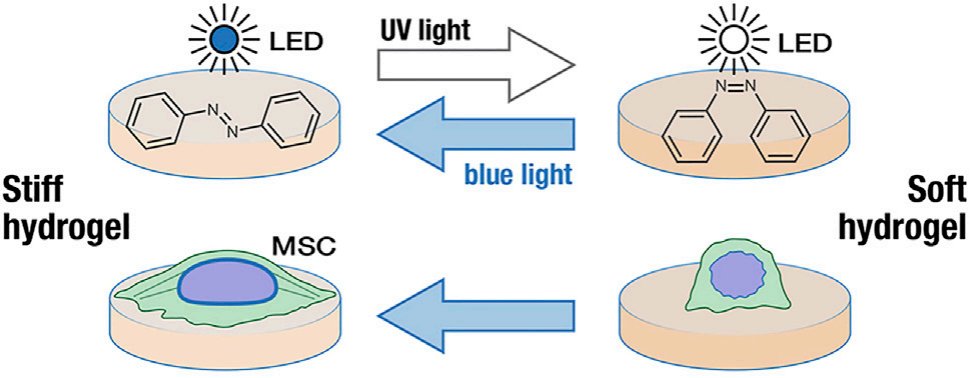
Changes of hydrogel stiffness under blue and UV light irradiation (Lee et al., 2018b). Reprinted (adapted) with permission from (Lee et al., 2018b).Copyright 2018–2021 American Chemical Society.

Stimulated responsive hydrogels can respond to light, heat, pH, magnetism, electricity, etc., causing changes in their stiffness (Abdollahiyan et al., 2020b). Photo-responsive hydrogels are usually composed of photo-responsive units and polymer networks. Under the action of light, photo-responsive units undergo isomerization, cracking or other reactions to convert optical signals into chemical signals, which changes the stiffness of hydrogels (Echeverria et al., 2018). And the optical signal, as a trigger in the photo-responsive hydrogels, can remotely control the self-structure of hydrogels and cause changes in mechanical properties (Burdick and Murphy, 2012). Compared with traditional stimulation conditions, the optical signal regulation has biocompatibility and “reagent-free” irritation (Richards et al., 2018). Lee et al. (2018b) designed a PAAm photo-switchable hydrogel based on photo-responsive molecule azobenzene. The photo-crosslinking agent is 4, 4′-bis (acrylamide) azobenzene (AZO), which can change from *trans*-structure to *cis*-structure under UV irradiation and blue light as well. In addition, Lee et al. analyzed the stiffness of hydrogels under different light irradiation. The results showed that the hydrogel was softened under UV light irradiation while the stiffness of the hydrogel was significantly improved by blue light irradiation (Figure 1). Unlike Lee’s method, Yuan et al. introduced IR780 near-infrared photothermal agent and calcium ions into methacrylic acid hyaluronic acid (MA-HA) hydrogels to prepare a three-layer hydrogel cell culture platform, in which the MA-HA layer was formed by cross-linking of solidum alginate (SA) and dithiothreitol (DTT). Under the irradiation of near-infrared light, the diffusion of phase change material (PCM) and calcium ions through the intermediate layer initiates the crosslinking of SA, resulting in a significant increase in the stiffness of the hydrogel (Yuan et al., 2021). What’s more, there are many studies on the stiffness of photo-responsive hydrogels. Poly (1-vinyl imidazole-co-methacrylic acid) hydrogels containing gold nanorods (AuNRs) exhibited improved mechanical properties under near-infrared light irradiation (Dai et al., 2020). Matrix stiffness and cell diffusion range of hydrogel with linear PPAm and photo-crosslinking agent decreased under UV irradiation (Frey and Wang, 2009). Rosales et al. (2017) designed a (HA) composite hydrogel, softened under 365 nm light irradiation, and restored matrix stiffness under 400–500 nm light irradiation.

In addition to light stimulation, the self-assembled hydrogels responding to pH also have improved mechanical properties. pH responsive hydrogels generally contain ionizable groups. With the change of environmental pH, they can accept protons or provide protons. Due to the change of pH environment, this charge change lead to the alternation of the hydrodynamic volume of the polymer chain, resulting in the change of the material between the collapse state and the expansion state (Gil and Hudson, 2004). Patra et al. (2010) designed a histidine hydrogel, and the imidazole side chain in the structure of histidine derivatives grants the hydrogel system pH-responsiveness. The experimental results showed that the gelation degree of hydrogels is different under different pH conditions, indicating that the change of pH led to the change of gel stiffness. King et al. (2016) designed a β-sheet peptide two-component self-assembly hydrogel and tested its rheological mechanics. The experimental results showed that too high or too low pH could lead to a rapid decrease in the stiffness of the hydrogel. At the same time, the stimulation change of temperature also caused the change of mechanical properties of hydrogels. The poly (N-isopropylacrylamide) (PNIPAAm) hydrogels prepared by Akimoto et al. (2018) showed good biocompatibility. When the temperature changed from 25°C to 37°C, they found that the stiffness of hydrogels gradually increased, showing improved mechanical properties. This hydrogel is characterized by the existence of a lower critical solution temperature (LCST). Due to its hydration/dehydration characteristics, the hydrogel at LCST reflects the transition coil-to-globule transition, resulting in expansion or contraction of its volume (Gil and Hudson, 2004). In addition, the effect of the magnetic field also has a certain influence on the stiffness of hydrogels. The magnetic hydrogel is obtained by adding magnetic particles into the hydrogel, so it can respond to the magnetic field. Abdeen et al. (2016) added carbonyl iron (CI) particles to the PA hydrogel, the hydrogel with stiffness transformation can be obtained by changing the magnetic field. At the same time, the author also used hydrogel to study the behavior of MSC cells. They found that matrix stiffness was related to cell morphology and differentiation (Abdeen et al., 2016).

### Preparation of Hydrogels With Different Stiffness by Changing the Molecular Weight of Materials

Some hydrogels can also adjust the stiffness by changing the molecular weight of materials, such as HA hydrogels mentioned above. Hyaluronic acid is a non-toxic, hydrophilic, and biodegradable polysaccharide (Graça et al., 2020; Hong et al., 2021), it is also one of the components of the cytoplasmic matrix, which is widely distributed in the human body (Burdick and Prestwich, 2011; Graça et al., 2020). Ren et al. (2021) reported HA hydrogels with different stiffness can be obtained by using HA of different molecular weights and grafting maleimide functional groups. And the experimental results show stiffness of the hydrogels varies greatly with the change of HA molecular weight. It is not only the increase of the molecular weight of HA, but also the physical entanglement and physical crosslinking in the main chain of HA that contribute to the change of the strength of the hydrogel. Moreover, this study showed that HA hydrogels with different molecular weights had different effects on genes and proteins related to cartilage differentiation. In addition, Kim et al. (2021) designed an Oxidized hyaluronate/glycol chitosan/adipic acid dihydrazide/hyaluronate–alginate hybrid (OHA/GC/ADH/HAH) hydrogel, which attempted to change the stiffness of the hydrogel by changing the molecular weight of the HA used to synthesize HAH. Under the condition of constant HAH concentration, the experimental results showed that with the increase of HA molecular weight (21, 200, and 735 KDa), the G′ of OHA/GC/ADH/HAH hydrogels also increased, and reached the peak when HA molecular weight was 735 KDa (Kim et al., 2021). Other studies have prepared methacrylhydrazide-HA (MAHA) hydrogel, its stiffness also increased with the increase of molecular weight of macromolecular monomer (Bobula et al., 2017). Apart from this, studies have also shown that keratin hydrogels with different molecular weights can affect the activity of wound healing and may be a good choice for tissue repair (Gao et al., 2020).

### Regulation of Hydrogel Stiffness by Changing the Different Proportions of Components

PAAm is one of the commonly used preparation materials for hydrogels, which is non-toxic and the stiffness of hydrogels can be tuned by changing the number of monomers of PAAm to change the stiffness of hydrogels (Kandow et al., 2007; Stanton et al., 2019b; Fang et al., 2019). For example, the cross-linking ratio of hydrogels using acrylamide and biacrylamide increases with the rise of the ratio of acrylamide to biacrylamide, resulting in higher stiffness hydrogels (Yang et al., 2016). At present, PAAm has also become one of the common stiffness adjustable hydrogel synthetic materials. Yang’s group prepared a biomimetic hydrogel with stiffness up to kPa through PAAm (Yang et al., 2016). Human plasma FN was used as the basis of cell adhesion, and the hydrogel was crosslinked with FN by UV light irradiation. Through the experimental data, we can get that PAAm hydrogel with different stiffness can be obtained by changing the content of the PAAm monomer. When the ratio of acrylamide/diacrylamide content gradually increased, both the crosslinking degree of the material and the stiffness were enhanced. At the same time, with the increase of matrix stiffness, the diffusion area of MSCs raised accordingly, suggesting that the behavior of MSCs changed with the variety of related biophysical clues. Sun et al. also obtained the hydrogel with adjustable stiffness (up to 32 kPa) by changing the content of acrylamide and crosslinking agent bisacrylamide (Sun et al., 2018). Among them, GO was used as the coating material of PAAm hydrogel. Finally, the GO/PAAm hydrogel composite scaffold was acquired. In addition, the multifunctional PAAm hydrogel can be obtained by 3D printing in a water environment, which also altered the stiffness of the material by changing the content of acrylamide and cross-linking agent bisacrylamide (Wang and Li, 2020). Considering the different preparation methods of PAAm hydrogel, we can find that the change of the ratio of PAAm monomer acrylamide to cross-linking agent acrylamide is the decisive factor to change the stiffness of PAAm hydrogel. Besides, Lokhande et al. (2018) combined κCA with silicate nanoparticles to form a composite hydrogel. The stiffness of the hydrogel raised significantly with the increase of the ratio of silicate nanoparticles to κCA (Lokhande et al., 2018). This is due to the charge on the surface of silicate particles, which enhances the electrostatic force between silicate particles and κCA, enhances the κCA network and increases the hydrogel stiffness.

### Regulating the Stiffness of Hydrogels by Adding Nanomaterials

Besides the methods above, the mechanical properties of hydrogels can also be improved by adding nanoparticles into hydrogels. The nanomaterials added into hydrogels include metal nanoparticles, carbon-based nanomaterials, ceramic nanoparticles, and so on (Chen et al., 2018; Bao et al., 2019a). Among them, nanoparticles can be incorporated into hydrogels through *in-situ* polymerization, *in situ* growth of the NPs, and physical mixing. Since nanoparticles can interact with polymers, such as hydrogen bonds and van der Waals interactions, the network of hydrogels can be strengthened to obtain nanocomposite hydrogels with stable structure and improved mechanical properties (Chen et al., 2018). GO is a carbon-based nanomaterial. Adding GO to hydrogel can improve the stiffness of hydrogel (Jo et al., 2017). In addition to GO, Cheng et al. (2018) dispersed HAP and whitlockite WH nanoparticles in different proportions into gelatin methacryloyl (GelMA) solution. And then they were crosslinked by ultraviolet light to form nanocomposite hydrogels. The mechanical properties of the hydrogel were characterized. It was found that the stiffness of the hydrogel increased with the increase of nanoparticle concentration, but decreased with the increase of WH content, indicating that different types of nanoparticles had different effects on the stiffness of the hydrogel. Huang et al. (2018) prepared nanocomposite hydrogels based on PVA, nano-hydroxyapatite (n-HA), and magnetic nanoparticles (Fe_2_O_3_). The final hydrogel was obtained by freeze-thaw operation and ultrasonic dispersion. Compared with pure PVA hydrogels, the mechanical properties of the hydrogels with nanoparticles were enhanced. Different freezing and thawing times may also change the compactness of the hydrogel network and thus change the stiffness of hydrogel. Kumar et al. (2018) prepared a mixed hydrogel network of GO, cellulose nanocrystals (CNCs), and polyacrylamide-sodium carboxymethyl cellulose (PMC). Because of the role of GO and CNC, and the same freezing and thawing operation, the mechanical properties of PMC-GO/CNCs hydrogels have been greatly improved, including stiffness (elastic modulus). In addition to homogeneous hydrogels, some studies also explored hydrogels with stiffness gradients. The gradient composite hydrogel can be obtained by adding nano silicate into GelMA and methacrylated kappa carrageenan (MκCA). Compared with pure hydrogel, the mechanical properties of the composite hydrogel are improved, and the cells showed different morphological distribution in the two media. Osteoblast morphology appears on the hard matrix GelMA-nSi, and cartilage cell round morphology appears on the soft matrix MκCA-nSi. Expected for cartilage and bone tissue engineering (Cross et al., 2018). At the same time, cells have different migration behaviors on such gradient hydrogels, and the function of cells (such as BMSCs) in corresponding parts after directional migration may promote tissue repair (Smith Callahan, 2018; Dekoninck and Blanpain, 2019; Fu et al., 2019; Motealleh and Kehr, 2020).

## Effects of Hydrogel Stiffness on Tissue Repair

Different synthesis methods can obtain hydrogels with different stiffness, and the different stiffness of hydrogels led to various cell behaviors. Different stiffness hydrogels can affect the behavior of stem cells, MSCs, fibroblasts (Ibañez et al., 2021), macrophages, and other cells, thereby affecting the process of tissue repair or bone defect. In particular, hydrogels affect the phenotype of macrophages through changes in stiffness (Lv et al., 2015), leading to changes in tissue inflammatory environment and angiogenesis, and ultimately promoting wound healing. How hydrogels affect the expression of related proteins or oxidative stress, leading to changes in cell behavior has become a research hotspot in recent years. Therefore, it is necessary to explore the mechanism of cell behavior changes caused by hydrogel stiffness changes applied to tissue repair and bone tissue regeneration. Here, we mainly discuss the effect of hydrogel stiffness change on MSCs and macrophages and its role in tissue repair.

### Effect of Hydrogel Stiffness on Stem Cell/MSCs Behavior

**FIGURE 2:**
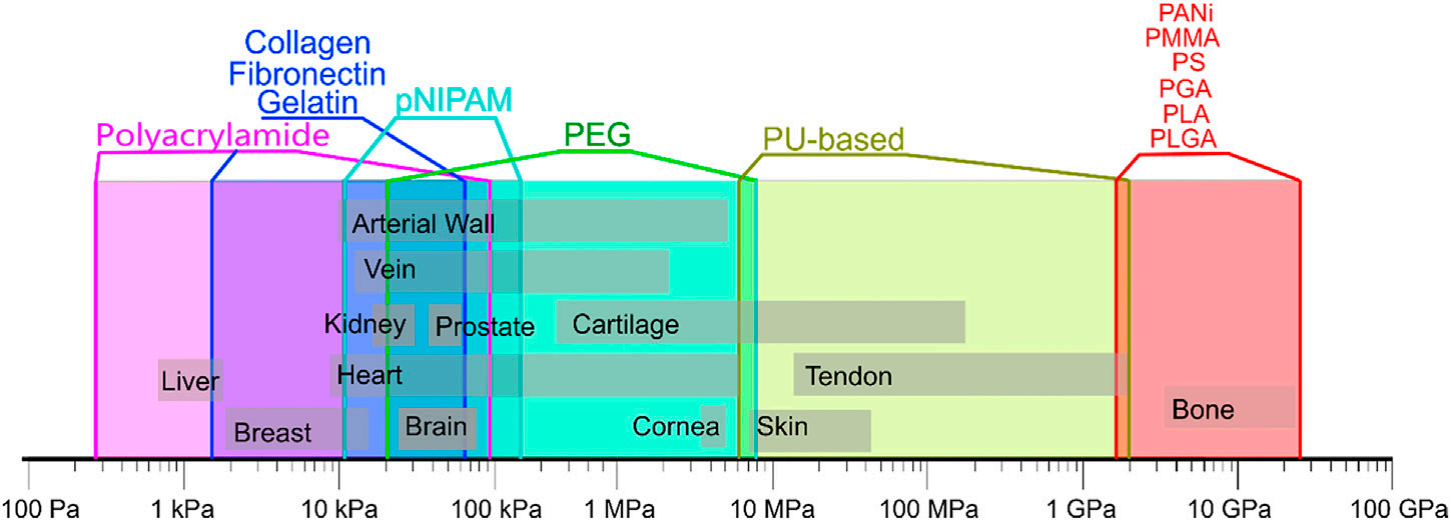
Mechanical properties of natural tissues and polymers. Data are composed based on data from the following publications (Nemir and West, 2010; Kim et al., 2012; Saveleva et al., 2019). Reprinted (adapted) with permission from (Abalymov et al., 2020).

The change of hydrogel stiffness affects the behaviors of cells (Kim et al., 2020), such as differentiation, migration and adhesion of cells, which has attracted wide attention of researchers. As for MSCs, for example, MSCs are likely to differentiate into osteoblasts under high matrix stiffness, while adipocytes appear with low matrix stiffness. MSCs are likely to differentiate into osteoblasts under high matrix stiffness, while adipocytes appear with low matrix stiffness. As introduced in the previous section, hydrogels have size-adjustable matrix stiffness and are degradable and non-toxic. Biophysical cues that mimic the extracellular matrix (ECM) through hydrogels can affect the fate of stem cell/MSCs. At the same time, the stiffness required by different tissues is different. Therefore, through the adjustment of materials, stem cells can be differentiated in specific directions, as shown in Figure 2 (Abalymov et al., 2020). For example, MSCs are likely to differentiate into osteoblasts in places with high-matrix stiffness, while adipocytes in places with low-matrix stiffness. In some studies, the expression of neurogenic proteins can also be observed on low stiffness hydrogels. In the experiment of Bi et al. (2019), a biocompatible hydrogel formed by cross-linking polyamidoamine (PAMAM) dendrimer and thiolated multi-armed polyethylene glycol (PEG) was designed. The stiffness of the hydrogel and the adhesion of MSCs to the hydrogel were improved by adjusting the concentration of the composite and adding the peptide sequence arginine-glycine-aspartate (RGD)derived from fibronectin. The hydrogel was used for osteogenic culture, and the fluorescence image of osteogenesis was obtained by laser scanning confocal microscope after phalloidin staining. The results showed that the hydrogel with high stiffness was more conducive to osteogenic differentiation than the hydrogel with low stiffness, and it was observed that the hydrogel with low stiffness had strong adipogenic differentiation ability. When hMSCs were cultured with gelatin-hydroxyphenylpropionic acid (Gtn-HPA) and their differentiation lineages were analyzed, the high stiffness hydrogel increased the amount of myogenic protein and the cell adhesion and proliferation area (Wang et al., 2010), indicating that matrix stiffness affects the direction of MSCs differentiation. In addition, other related behaviors of MSC are also affected by matrix stiffness. Studies have shown that MSC tends to migrate from softer substrates to harder ones and diffuse more on harder ones (Kim et al., 2020). At the same time, through the fluorescence staining results of Liu et al., (2020), we can find that MSC has stronger cell adhesion and cell viability on the hydrogel with higher stiffness. What’s more, in the experiment of Zhan, MSCs proliferated better and had better cell viability in hydrogels with higher stiffness (Zhan, 2020). It is well known that cells need to have good migration, adhesion, differentiation and other behaviors on hydrogels to facilitate the synthesis of extracellular matrix components and thus facilitate tissue repair (Vainieri et al., 2020). Therefore, the cell behavior can be regulated by changing the stiffness of hydrogels, so that the cells can function well in the corresponding parts.

In the process of the above hydrogel stiffness affecting cell behavior, it may regulate cell expression by affecting the expression of related genes and proteins (Labouesse et al., 2021). In human adipose-derived stem cells (hADSCs) encapsulated by methacrylated hyaluronan (MeHA) hydrogel, the contents of two cartilage-related gene aggrecans and type II collagen increased significantly with the increase of hydrogel stiffness. At the same time, s-GAG and type II collagen increased, more collagen cartilage formed, hADSCs differentiated into cartilage (Teong et al., 2018). In the process of differentiation, the increase of alkaline phosphatase content is also reflected in the trend of osteogenic differentiation of stem cells with the increase of matrix stiffness (Darnell et al., 2018). In the study of Duan et al. (2019), MSCs express more genes such as Rac1, RhoA and CD44 that promote mesenchymal migration in high stiffness hydrogels. At the same time, high expression of CD44 also affects cell migration, which plays a vital role in wound healing (Zhu et al., 2006). What’s more, Murphy et al. (2017) found that the hydrogel with higher stiffness has more Vascular endothelial growth factor (VEGF) expression, which can promote wound healing by regulating neovascularization. Simultaneously, platelet derived growth factor (PDGF) had more secretion on the surface of hydrogels, which played a key role in the inflammatory response stage of wound healing (Murphy et al., 2017). In addition, studies have shown that the transmission of hydrogel stiffness may be affected by the signal transmission of related proteins. SORBS1 adhesion spot protein affects the differentiation pedigree of MSCs. In the process of hydrogel culture of MSCs, the knockdown of SORBS1 adhesion spot protein reduced the nuclear expression of CBFA1 and Osterix by more than 50% (Holle et al., 2016). Cell activity and phenotype were well maintained in hydrogels with high stiffness (Thomas et al., 2017). Lee et al. pointed out that the osteogenic signal runx2 and osteopontin expression of MSCs raised with the increase of hydrogel stiffness (soft ∼0.5 kPa or hard ∼40 kPa), and MSCs grew faster on hydrogels with higher matrix stiffness. In the experiment, they found that, like other studies (Yang et al., 2014), hydrogels with higher matrix stiffness may lead to some irreversible activation. When cells move from hard matrix to soft matrix, the osteogenic marker runx2 did not decrease significantly (Lee et al., 2014).

**FIGURE 3:**
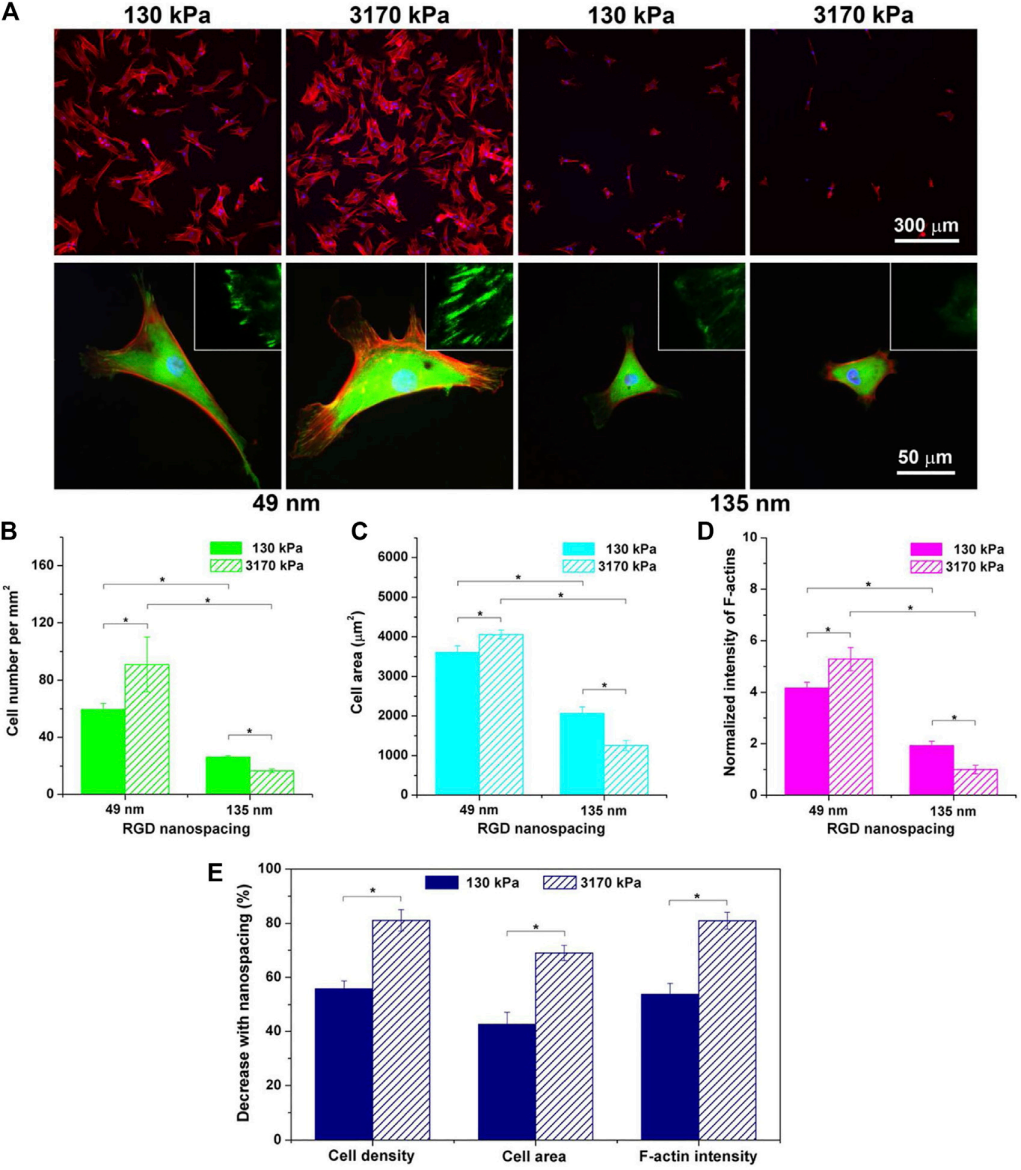
Fluorescence images of cells on RGD-nanopatterned PEG hydrogels and corresponding statistical results as functions of hydrogel stiffness and RGD nanospacing. **(A)** Cells were cultured on RGD nanopatterns for 24 h and stained for observations of vinculins (green), F-actins (red), and nuclei (blue). The upper and the lower rows show typical low-magnification and high-magnification fluorescence micrographs, respectively. The insets in the lower row demonstrate further magnified local images to show vinculins around the periphery of cells. **(B–D)** Statistical results of cell density **(B)**, cell spreading area **(C)**, and fluorescent intensity of F-actins normalized to the mean value of the group of the stiff hydrogels (3,170 kPa) with the large RGD nanospacing (135 nm) **(D)**. Mean values and standard deviations from three independent experiments are presented. **(E)** Dependence of the indicated parameters upon RGD nanospacing as characterized by the decline percentage at a given matrix stiffness calculated with Eq. 1 (*n* = 3 × 3). “*”: *p* < 0.05 in a student’s t-test, indicating a significant difference between the two groups. (Ye et al., 2015). Reprinted (adapted) with permission from (Ye et al., 2015). Copyright (2015–2021) American Chemical Society.

The stiffness of hydrogels does not simply directly affect the gene expression of cells in proteins. As one of the components of the assembled cytoskeleton, stress fibers felt and transferred stiffness changes from the external environment during cell growth. And the increase in stiffness can increase the number of stress fibers. Bao et al. (2019b) cultured hMSCs with 3D micropores prepared by MeHA hydrogel as the matrix. In the cells with the largest volume, the value of matrix stiffness was from small to large and the stress fibers were from none to gradually enhance. Finally, cells with a clear cytoskeleton could be seen in the hard hydrogel. Description of hydrogel stiffness is a key factor affecting MSCs behavior (Bao et al., 2019b). Interestingly, Park et al. (2011) proposed that when MSCs were cultured in hydrogels with different stiffness, hydrogels with weak stiffness (soft materials) might reduce the formation of stress fibers and regulate the expression of proteins related to the formation of cartilage and adipocytes by inhibiting the behavior of Rho-induced stress fibers, resulting in differences in MSCs morphology and differentiation. Ye’s group (Ye et al., 2015) tested the morphological differentiation characteristics of MSCs on substrates with different RGD peptide spacing and different hydrogel stiffness. As shown in Figure 3, after fluorescence staining, they found that on hydrogels with small RGD nano-spacing, hydrogels with high stiffness had stronger adhesion spots and clearer stress fibers. In addition, the small RGD hard hydrogel has a stronger F-actin.

**FIGURE 4:**
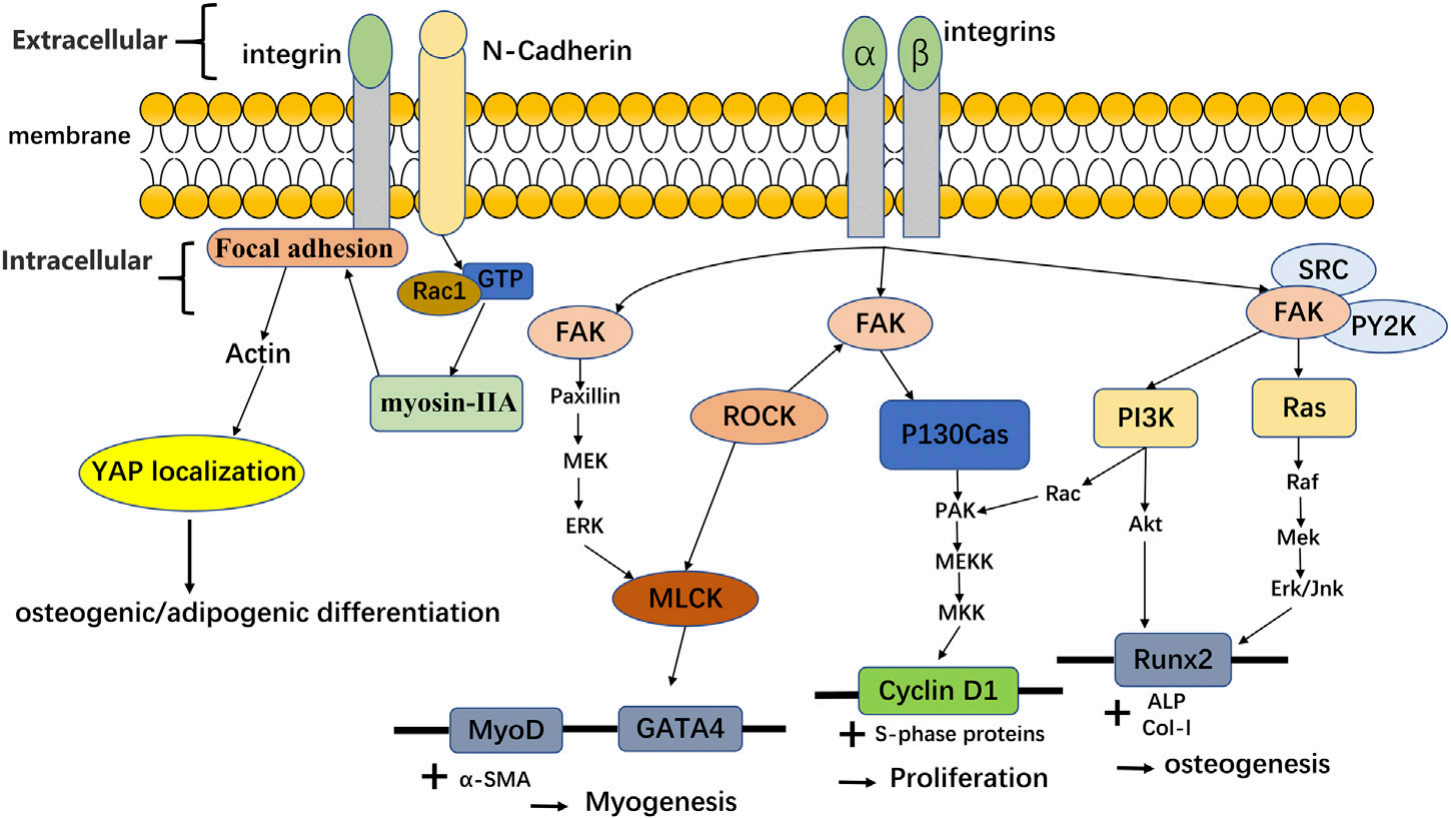
Integrins and cadherin affect the differentiation and proliferation of MSCs by affecting the related protein/YAP translocation. Adapted with permission from (Selig et al., 2020). Related data adapted from (Cosgrove et al., 2016).

The change of hydrogel stiffness for cell response is also related to the mechanical transmission of YAP/TAZ. The nuclear YAP is a mechanically sensitive protein. The change of matrix stiffness can regulate the expression of transcription factors YAP and TAZ. On the substrate with high stiffness, YAP/TAZ nucleus accumulates through Runx2 to guide cell osteogenic differentiation. On the substrate with low stiffness, YAP/TAZ translocation to cytoplasm can also lead cells to adipogenic differentiation, so YAP and TAZ can induce the fate of MSCs differentiation (Chen et al., 2016; Driscoll et al., 2015; Zhang et al., 2021). After depletion of YAP and TAZ, osteogenic differentiation of cells was inhibited. And the knockdown of YAP and TAZ made it for MSCs to be induced into adipocytes on the hard matrix. These indicate that stiffness may affect stem cell lineage selection through YAP and TAZ (Dupont et al., 2011). In another experiment (Ren et al., 2021), the hydrogel with low mechanical strength can express more dry genes than the hydrogel with high strength through the classical Wnt pathway to maintain cell dryness (self-renewal ability). Hydrogels with high mechanical strength can affect calcium influx through transient receptor potential vanillin 4 (TRPV4), thereby affecting cell differentiation into cartilage. In addition, studies have shown that the interaction between cadherin and integrin can affect the relevant mechanical conduction and thus affect the YAP/TAZ nuclear localization, making MSCs differentiate towards various lineages (Zhang et al., 2021; Cosgrove et al., 2016). For integrins, by staining α2-integrins on hydrogels with different stiffness, it was found that the higher the matrix stiffness was, the more α2-integrins were expressed by cells. The changes in the number of integrins can indirectly affect nuclear focal adhesion kinase (FAK) by sensing the changes in stiffness. And the activation of FAK can also affect ERK1/2, thus affecting a series of procedures of MSC osteogenic differentiation (Shih et al., 2011). After FAK activation, it can also activate phosphoinositide 3-kinase (PI3K) or pile protein, thereby triggering the conduction of downstream signals, and then enhancing MSCs proliferation, myogenic differentiation, or osteogenic differentiation. Besides, integrins/cadherin can also enhance the proliferation or differentiation of MSCs by affecting other proteins or YAP, as shown in Figure 4. In the mechanical conduction process of YAP/TAZ, Caveolin-1 can be used as the upstream signal to regulate the downstream signal conduction of YAP/TAZ. Caveolin can mediate the change of substrate stiffness (Moreno-Vicente et al., 2018). Xiang et al. found that the expression of Caveolin-1 on soft gel was lower than that on hard gel when hBMSC was cultured with soft/hard gel. At the same time, the experimental results also showed that the low level of Caveolin-1 expression cause the high expression of YAP/TAZ, which result in the subsequent regulation of YAP/TAZ enhanced the fat generation ability of hBMSC (Xiang et al., 2021). In addition to integrin and Caveolin, Pizeo can also perceive mechanical conduction and influence YAP nuclear localization (Emig et al., 2021). Piezo is a cation channel that can sense mechanical force, including Piezo1 and Piezo2 (Sun et al., 2020). Piezo1, as a mechanical force receptor, can respond to mechanical stimuli. In the experiments of Wang et al. (2020b), they found that Piezo1 could perceive mechanical load, and Piezo1 could regulate the expression of type II and type IX collagens by changing the nuclear localization of YAP, thus affecting the osteogenic process. In addition, the expression of Piezo1 increased with the rise of gel stiffness in the external environment (Sun et al., 2020; Fu et al., 2021). The related experiments carried out by Sun et al. with human umbilical cord mesenchymal stem cells (hUC-MSCs) revealed the interaction among Piezo1, integrin and Ca^2+^. The results shown that integrins interacted with the cytoskeleton, and structural changes in the cytoskeleton inhibited Piezo1 channels, resulting in less Ca2 + entering the cytoplasm. Moreover, Piezo1 with different stiffness has obvious dependent growth behavior (Sun et al., 2020). At present, many studies in Piezo1 channel focus on its influence on neural stem cells (Pathak et al., 2014), osteoblasts (Li et al., 2019) and so on, but few studies was found on MSCs.If the mechanism of Piezo1-integrin-FAK and other related mechanisms can be clearly understand and found, it will promote the effect of extracellular environment changes on tissue repair.

### Effect of Hydrogel Stiffness on ROS Expression for Angiogenesis

**FIGURE 5:**
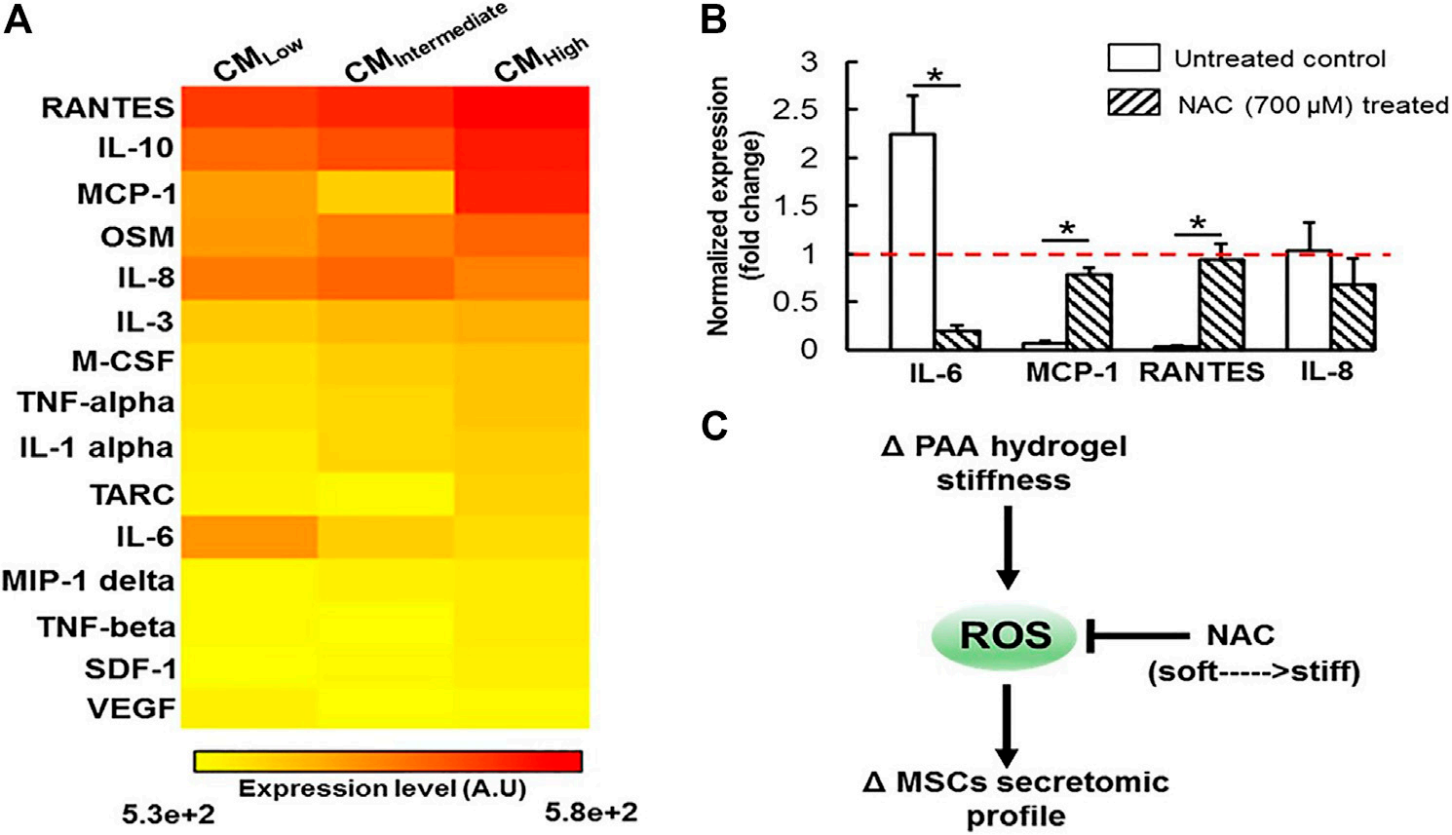
Substrate stiffness modulates MSCs secretomic landscape via a ROS dependent mechanism. **(A)** Heatmap analysis of 15 secreted proteins by MSCs revealed substrate stiffness is a potent regulator of MSCs secretome. **(B)** The impact of NAC treatment on expression of several mechanosensitive secreted proteins was assessed at the transcript level, which is normalized to the level of the housekeeping 18s gene (dashed horizontal line). NAC treatment significantly altered expression of IL-6, MCP-1 and RANTES in a manner that is similar to culturing the cells on substrate with different stiffness, suggesting that ROS may play an important role in mediating the substrate stiffness effect. All data are presented as mean ± SD (*n* = 5) * denotes statistical difference at *p* < 0.05. **(C)** Schematic diagram depicting the proposed mechanism by which substrate stiffness can alter MSCs secretome in a ROS-dependent manner. Reprinted (adapted) with permission from (Yang et al., 2016). Copyright (2016–2021) American Chemical Society.

ROS is a general term for a class of oxygen-containing active chemicals in the body, including singlet oxygen (^1^O_2_), hydrogen peroxide (H_2_O_2_), hydroxyl radical (OH·), etc., and ROS certainly plays a role in maintaining related physiological functions in the body. Excessive ROS can lead to oxidative stress (Hayyan et al., 2016; Nosaka and Nosaka, 2017; Yang et al., 2019b; Li et al., 2016). As mentioned in the previous section, the change of hydrogel stiffness was mediated by some mechanically sensitive proteins Rho and YAP. In the experiment of stiffness-induced MSCs behavior changes, ROS can act as upstream signal to regulate the behavior of these protein secretion groups. (Yang et al., 2016). ROS of MSCs grown on different hydrogels was evaluated by fluorescence living cell microscope and the conclusion was that ROS expression decreased with the increase of hydrogel stiffness. Surprisingly, with the increase of ROS expression on weak hydrogels, cells showed non-toxic expression. As we all know, the proteins secreted by BMSC, including IL-8, monocyte chemoattractant protein-1 (MCP-1), regulated upon activation, normal T-cell expressed and secreted (RANTES), and type 1 collagen, are important substances involved in tissue repair. The changes in hydrogel stiffness affect the expression of ROS, thereby affecting the expression of related secretory factors and thus playing a role in tissue repair (Yang et al., 2016). Figure 5 shows that matrix stiffness regulates MSCs secretion group in ROS-dependent manner. Another study also showed that with the decrease of hydrogel matrix stiffness, the content of ROS detected by CellRox Orange increased, indicating that human adipose-derived MSC (ADMSC) was in a state of oxidative stress (Yang et al., 2019c). By testing the total antioxidant capacity (TAC), it was found that the content of TAC increased with the decrease of hydrogel stiffness, Indicating that the redox metabolism of ADMSCs was affected by substrate stiffness. In addition, the experiment also showed that hydrogel stiffness can be used for tissue repair by indirectly affecting the expression of VEGF. In the experiment, the conditioned medium (CM) of ADMSC ultured on different stiffness hydrogels was used to treat human umbilical vein endothelial cells (HUVEC). It was found that the hydrogel with the smallest stiffness enhanced the angiogenesis ability of endothelial cells. The process is that the decrease of hydrogel stiffness affects the expression of HIF1α signal by affecting the change of ROS expression, thereby affecting the expression of VEGF cytokines and then affecting the angiogenesis of HUVEC (Yang et al., 2019c). In addition, Lee et al. (2017) also found that after HMSC differentiated into endothelial cells, stronger angiogenesis was observed on hydrogels with higher matrix stiffness.

### Effects of Hydrogel Stiffness on Immune Response

In regenerative medicine and tissue engineering, scaffold materials are often used to repair related tissues. When the scaffold is implanted into the defected tissue, the immune response and inflammatory response are the initial manifestations of tissue healing. How to regulate immune/inflammatory response to improve the application of scaffold materials in tissue repair has become a research hotspot (Badylak and Gilbert, 2008; Spiller et al., 2015; Alvarez et al., 2016). Macrophages play a significant role in immune/inflammatory response and the stiffness cells/MSCs. In addition, as stiffness affects stem cells/MSCs, changes in hydrogel stiffness can also lead to the expression of different proteins and cytokines in macrophages and the nuclear localization of YAP, thus playing a role in tissue repair. Therefore, we can improve the immune/inflammatory response by changing the stiffness of hydrogels to affect the phenotype of macrophages. The cell diffusion area and short axis length of cultured macrophages indicated by different stiffness hydrogels were stronger than those of soft hydrogels by dead staining (Zhuang et al., 2020). With the increase of matrix stiffness, F-actin was more widely dispersed, and the content of focal adhesion (FA) increased. In addition, by observing the inducible nitric oxide synthase (iNOS) and arginase (Arg-1) expression of macrophages, it was found that iNOS was expressed more while Arg-1 was decreased on the hard hydrogel. Consistent with other studies (Okamoto et al., 2018; Sridharan et al., 2019a), the hard hydrogel caused the conversion of macrophages to M1 (pro-inflammatory) macrophages. Thus, the expression of related pro-inflammatory factors such as Tumor necrosis factor-α (TNF-α) increased (Zhuang et al., 2020). In general, the inflammatory response increases with the number of macrophages, and this experiment showed that increased macrophages mediate a less intense inflammatory response. In the experiment of Previtera and Sengupta (Previtera and Sengupta, 2015), the effect of hydrogel stiffness on the phenotype of stimulated macrophages was investigated. It was found that macrophages stimulated by lipopolysaccharide (LPS) or pro-inflammatory factor TNF-α also increased the secretion of pro-inflammatory factors such as TNF-α, IL-1β, and IL-6 as the matrix stiffness became stronger. In addition, when the western blotting analysis was performed on the lysates of bone marrow-derived macrophages (BMM), the toll–like receptor 4 (TLR4) produced by BMM increased with the increase of hydrogel stiffness (0.3 and 230 kPa), indicating that LPS was bound to the increased TLR4 under high stiffness, resulting in more secretion of pro-inflammatory factors.

After the scaffold material was implanted into the tissue, the phagocytosis of macrophages plays an important role in controlling inflammatory response (Bessa-Gonçalves et al., 2020; Mahon et al., 2020; Yang et al., 2020). Macrophages can phagocytize apoptotic cells and eliminate pathogenic microorganisms, which is more conducive to the implantation of scaffold materials. When the stiffness of the hydrogel matrix increases, although it is introduced that the diffusion area of macrophages is larger, its phagocytosis is contrary to the change of hydrogel stiffness, which decreases with the increase of hydrogel stiffness. And at the same time, phagocytosis is strongest in hydrogels with a medium stiffness (Sridharan et al., 2019b; Li and Bratlie, 2021). In addition to the sensory mechanical signals introduced in the previous section that affect the lineage selection of MSCs. YAP can also affect the behavior of macrophages. In hydrogels with higher stiffness, LPS induced more TNF-α and more nuclear localization of YAP (Freytes et al., 2013). Knockdown of YAP by siRNA can be found to reduce the expression of TNF-α in hydrogels, indicating that YAP is related to the enhanced inflammatory response. Soft/hard hydrogel was implanted into mice respectively. It was found that soft hydrogel affects the size of the wound compared to the controls, indicating that stiffness-mediated inflammatory response played a certain role in tissue repair (Meli et al., 2020). BMSCs are vital in tissue repair, which can differentiate into a variety of cell types. After the phenotypic changes of macrophages under different hydrogel stiffness, it changes between M1 and M2 types. Different types of macrophages secrete different cytokines. M1 pro-inflammatory type inhibited the proliferation of hMSCs, while the M2 type improved the proliferation of hMSCs (Freytes et al., 2013; Zhu et al., 2020).

**FIGURE 6:**
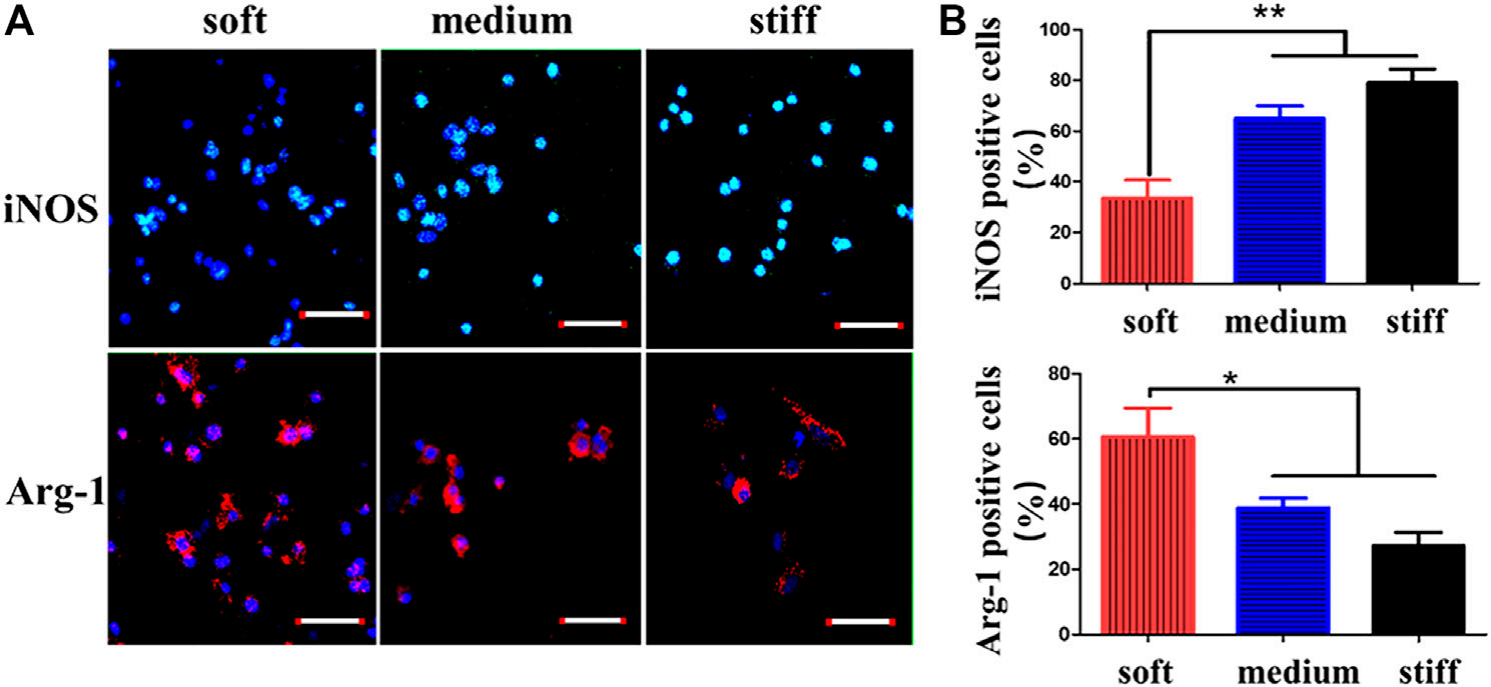
CD marker expression of macrophages on GelMA hydrogels of different stiffnesses. **(A)** Macrophages of GelMA hydrogels of different stiffnesses stained with iNOS (M1 marker) and Arg-1 (M2 marker). **(B)** Percentages of iNOS- and Arg-1-positive macrophages quantified after 3 days of macrophage culture. Reprinted (adapted) with permission from Zhuang et al., 2020). Copyright (2020–2021) American Chemical Society.

Angiogenesis is also an important process of stimulating repair in the process of tissue repair and bone healing. And the phenotype change of macrophages leads to angiogenesis. In the early stage of wound healing, M1 macrophages play a main role. And in the later stage, M2 macrophages replace M1 macrophages. VEGF is an initiating factor in angiogenesis. TNF-α and IL1-β produced by M1 macrophages under the influence of stiffness stimulate endothelial cells to produce VEGF and promote the early stage of wound healing. Cytokines produced by M2 macrophages (PDGF-BB) play a stabilizing role in late angiogenesis (Spiller et al., 2014; Spiller et al., 2015; Champagne et al., 2002; Tous et al., 2012). Therefore, the phenotype of macrophages can be changed by changing the stiffness of the hydrogel, so that macrophages play a certain role in tissue repair. Figure 6 shows the changes in the content of surface markers of M1 and M2 macrophages under different stiffnesses, indicating that the phenotype of macrophages changes with the change of stiffness. What’s more, the different proportions of M1 and M2 also lead to different levels of angiogenesis. Recently, in the experiment of Ji et al. (2020), BMSCs were carried by poly (*ε*-caprolactone) modified (PCL) chitin hydrogel with good mechanical strength. Under inflammatory conditions, BMSCs promoted the transformation of M2 macrophages and inhibited the expression of M1 macrophages-related factors, thereby promoting tissue repair. On the hydrogel platform with dynamic stiffness change, it is also reflected that high stiffness makes M2 type turn to M1 type and has a higher YAP/TAZ nuclear ratio. In addition, a higher YAP/TAZ nuclear ratio is also related to the osteogenic differentiation of BMSCs (Mosqueira et al., 2014). Therefore, in tissue repair or osteogenic effect, hydrogel stiffness may affect macrophage phenotype and BMSCs differentiation by affecting YAP/TAZ nuclear ratio, which affects related inflammatory and osteogenic processes to promote tissue repair.

In addition, the change of matrix stiffness affects the expression of ROS, and ROS as an upstream signal affects the nuclear factor-κB (NF-κB) pathway, causing the conversion of macrophages between M1 and M2 (Chen et al., 2020). At the same time, under the combined action of LPS and adenosine triphosphate, the content of mitochondrial ROS (mtROS) on the soft matrix increased (Chuang et al., 2020). The authors also showed that macrophages growing on the soft gel could produce more ROS under the pressure of LPS/adenosine triphosphate (ATP). And the increase of ROS could trigger inflammatory reactions (Bulua et al., 2011). Therefore, if the stiffness of the matrix increased, the decrease of ROS production would lead to the weakening of inflammatory reactions. It indicated that the changes of macrophage function caused by matrix stiffness were associated with ROS expression. Although we do not know the specific relationship between ROS and macrophages, we can control the inflammatory response by regulating the stiffness of the matrix.

## Prospective and Conclusion

Hydrogel is a bio-scaffold material with biocompatibility and biodegradability. The stiffness of hydrogel can be adjusted by changing the crosslinking mode, external stimulation, molecular weight, concentration ratio of composite materials, and adding nanoparticles. Hydrogels with different stiffness are promising materials in biomedicine and their stiffness will change the behavior of BMSCs. The higher hardness hydrogel can induce BMSCs to differentiate into osteoblasts while lower hardness hydrogel can induce BMSCs to differentiate into chondrocytes or adipocytes. Recently, a composite hydrogel with nano-silicate was developed. The gradient stiffness of hydrogel can change the morphology and behavior of cells, which has a certain effect on the tissue repair of the cartilage-bone interface. Recent studies have also found that hydrogels with physiological stiffness may also alter the behavior of corresponding cells by affecting the production of oxidative stress ROS, thereby playing a role in tissue repair or inflammatory response.

But there are still some problems limiting the application of hydrogels. For instance, the systematic toxicity of added nanomaterials still requires further clinical research. Besides, How the stimuli-responsive hydrogels should be effectively applied to the actual treatment by changing the external conditions is also a problem to be considered in the future. In addition, through in-depth exploration of the relationship between matrix stiffness and ROS, a new hydrogel material may be designed to improve the skin aging process by affecting the production of ROS in the future. At the same time, Combined with their drug-loading function (Falcone and Kraatz, 2018), hydrogels can have an enhanced repair effect. And with the advantage of low cost, they can be widely used for tissue defects.

In addition, the hydrogel types discussed above are carried out in 3D environment, but most of them are carried out in two-dimensional (2D) environment. We all know that the real environment of organ is a complex 3D structure, and it is difficult to obtain the fiber structure characteristics of the real extracellular matrix under 2D conditions. Therefore, the influence of stiffness on cells may be different in 3D or 2D condition. Recently, researchers have designed 3D hydrogels with adjustable stiffness to mimic the natural tissue environment. It’s found that the increase of 3D scaffold stiffness also promotes the proliferation and cartilage differentiation of BMSC, and the change of stiffness also affects the adhesion, migration and viability of cells (Hogrebe et al., 2018; Liu et al., 2020; Zhan, 2020). This indicates that in 3D environment, extracellular physical cues still have similar effects on cell behavior. What’s more, new studies have also used 3D hydrogels to simulate skin dermal and muscle tissues (Bacakova et al., 2019; Urciuolo et al., 2020). Among them, the change of hydrogel stiffness supports the correlation and affect the behavior of related cells.

In summary, hydrogels with different stiffness can affect the morphology or lineage selection of stem cells/MSCs and macrophages, thereby promoting or inhibiting tissue repair. In the future, researchers can prepare stiffness-adjustable hydrogels for tissue engineering by different methods and use changes in hydrogel stiffness to influence cell lineage selection to promote osteogenic differentiation or angiogenesis. This idea of regulating the cell microenvironment for tissue repair provides a promising strategy for tissue engineering and regenerative medicine.
